# Radioiodine Labeled Anti-MIF McAb: A Potential Agent for Inflammation Imaging

**DOI:** 10.1155/2007/50180

**Published:** 2007-10-18

**Authors:** Chao Zhang, Gui-hua Hou, Jian-kui Han, Jing Song, Ting Liang

**Affiliations:** ^1^Department of Nuclear Medicine, Qilu Hospital of Shandong University, Jinan 250012, China; ^2^Institute of Experimental Nuclear Medicine, School of Medicine, Shandong University, Jinan 250012, China

## Abstract

Macrophage migration inhibitory factor (MIF) is a proinflammatory cytokine that may play a role in the pathogenesis of inflammation. Radiolabeled anti-MIF McAb can be used to detect in vivo inflammatory changes. The objective of this study was to investigate in vivo biology of radioiodinated anti-MIF McAb using the inflammation model mice. Anti-MIF McAb was radioiodinated with NaI125 by Iodogen method. Animal models were induced in the mice by intramuscular injection of *S. aureus*, *E. coli*, and turpentine oil. The biodistribution studies with radioiodinated anti-MIF McAb were performed on inflammation mice. The relationship between inflammatory lesions and anti-MIF McAb binding was investigated using the percent of injected dose per gram tissue (% ID/g) of tissue samples and whole-body autoradiography. The radioactivity of I125-anti-MIF McAb in the inflammatory tissue increased gradually for three inflammation models. The highest uptake was found in *S. aureus* group and the lowest was in *E. coli* group. The uptake in turpentine oil group was average. Whole-body autoradiography showed that all inflammation foci could be visualized clearly from 24 hours after injection, but 48 hours images were much clearer in accordance with the high T/NT ratio. These results demonstrate the ability of radioiodinated anti-MIF McAb to measure in vivo inflammatory events represented by high expression of MIF and suggests that radiolabeled anti-MIF McAb warrants further investigation as a potential inflammation-seeking agent for imaging to detect inflammatory disorders.

## 1. INTRODUCTION

Macrophage migration inhibitor factor (MIF) was originally discovered as a kind of lymphokines involved in delayed type hypersensitivity and various macrophage functions [1–3]. However, its descriptive name was shown to be rather imprecise as MIF can also promote macrophage rolling and transmigration by upregulating P-selection expression in endothelial cells lining the site of inflammation [4, 5]. Numerous animal studies have revealed the critical role of MIF in acute and chronic inflammation [6, 7]. The increased levels of MIF in certain pathological conditions may be indicative of its involvement in those diseases. Indeed, increased MIF plasma or serum levels were identified in patients with severe sepsis [8], Crohn's disease and ulcerative colitis [9], acute pancreatitis [10], rheumatoid arthritis (RA) [11], type 2 diabetes (T2D) [12], Guillain-Barre syndrome [13], or multiple sclerosis [14]. Consequently, MIF's activity has become a potential target for treating these various disorders. In this study, we labeled anti-MIF monoclonal antibody (McAb) with radioiodine Na^125^I and investigated its biodistribution and pharmacokinetics in vivo in animal models with inflammation.

## 2. MATERIALS AND METHODS

### 2.1. Radioiodination of anti-MIF McAb

All commercially available chemicals were of analytic grade and anti-MIF McAb (R&D Systems) was pharmaceutical grade. Anti-MIF McAb was iodinated with Na^125^I (specific activity 37 MBq/mg, China Institute of Atomic Energy) using the Iodogen technique (Pierce). Radioiodinated antibody was separated from free iodine using a size exclusion column (Sephadex G-25, Pharmacia). The specific activity of radioiodinated antibody is 29.56 GBq/*μ*mol. The radiochemical purity is >95% (paper chromatography).

### 2.2. Preparation of inflammation animal model

The animal experiments were carried out in accordance with institutional, national, and international guidelines for humane use of animals for research. Fourty eight mice (BALB/c, 18 ∼ 22 g, Animal Center of Shandong University) were divided into three groups, each group consisting of 16 mice, respectively. The first and second groups were induced inflammation by intramuscularly injecting $2\times 10^{7}$–$10^{8}$ colony forming units (CFU) of *S. aureus* and *E. coli* in 0.2mL, respectively, into the left thigh muscle [15]. The third group of mice were induced sterile inflammation by intramuscular injection of 0.2 mL turpentine oil [15]. Twenty four hours after inoculation, focal inflammation occurred. Those inflammation models were proved by histological studies (data not showed).

### 2.3. Biodistribution of ^**125**^I-anti-MIF McAb

Mice with the left thigh inflammation were intraperitoneally injected with 3.7 MBq ^125^I-anti-MIF McAb in 0.2 mL PBS. Three mice of each group were sacrificed by cervical dislocation at 30 minutes, 4 hours, 24 hours, 48 hours, and 72 hours after injection, respectively. A sample of 1 mL blood was collected at the time of decapitation. Samples of two thigh muscles (left as target, right as control), lungs, heart, liver, spleen, kidney, and bone were removed and weighted. The tissue radioactivity was measured with a wipe test counter (CAPRAC). The percent of injected dose per gram tissue (% ID/g) was calculated by comparison with samples to standard dilutions of the initial dose.

### 2.4. Whole-body autoradiography

Three groups of mice inflammatory models were established by the same method like biodistribution study. Each group consists of 4 mice. ^125^I-anti-MIF McAb (3.7 MBq in 0.2 mL PBS) were injected intravenously via the tail vein. Serial images were performed at 24 hours, 48 hours, and 72 hours after injection. The anesthetized mice were placed on the storage-phosphor screen plate with the ventral side facing the plate, in subdued light. The plate was exposed to a mouse for 45 minutes. At cessation of exposure, the plate was immediately covered with an opaque plastic sheet, then transferred to the scanner, and scanned by typhoon trio + (laser red 633 nm, pixel size 200 mcrons, phosphor mode: best sensitivity).

### 2.5. Statistics

Dates were expressed as the $\bar{x}\pm \text{s}$. The dates were analyzed using SPSS11.0 software.

## 3. RESULTS

### 3.1. Antibody clearance from the blood

**Figure 1:**
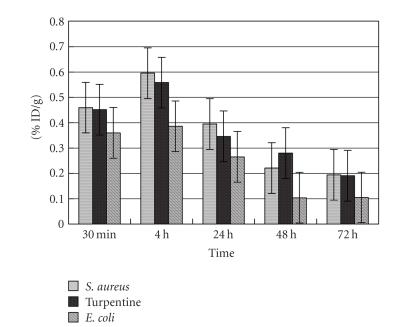
Clearance of ^125^I-anti-MIF McAb from the blood in *S. aureus* group, *E. coli* group, and turpentine oil group (% ID/g, $\bar{x}\pm \text{s}$).

^125^I-anti-MIF McAb shortly transited from the peritoneal cavity to the circulation after intraperitoneal injection. At first 30 minutes, the activity of ^125^I-anti-MIF McAb in the blood increased rapidly up to 45.00% ID, 36.66% ID, and 45.66% ID, respectively, in *S. aureus*, *E. coli*, and turpentine group. Then, it went up and reached a zenith at 4 hours post injection. Levels were 60.03% ID, 38.59% ID, and 54.42% ID, respectively, in three groups. After that point, the activity in the blood went down quickly (Figure 1).

### 3.2. Accumulation in the inflammatory tissue

**Figure 2:**
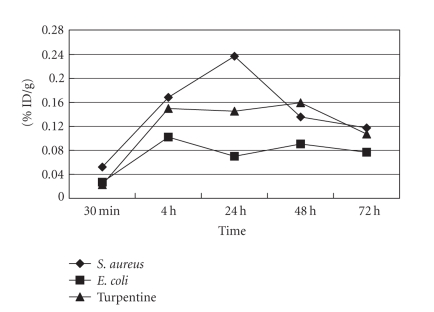
Accumulation of ^125^I-anti-MIF McAb in the inflammatory tissue of *S. aureus* group, *E. coli* group, and turpentine oil group (% ID/g, $\bar{x}\pm \text{s}$).

**Figure 3:**
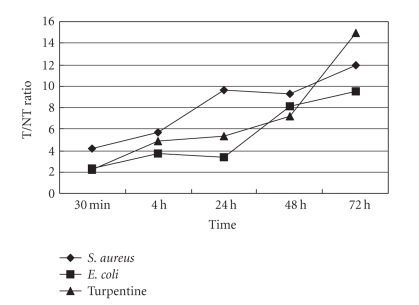
Change of T/NT in the *S. aureus*, *E. coli*, and turpentine oil group.

The concentration of ^125^I-anti-MIF McAb in the inflammatory tissue was expressed as a percentage of the initial dose (% ID/g, Figure 2) and T/NT (target/nontarget) ratio (Figure 3). The activity of ^125^I-anti-MIF McAb in the inflammatory tissue increased gradually for three inflammation models. The highest uptake was *S. aureus* group and the lowest was *E. coli* group. The uptake of turpentine oil group was average. In all three groups, T/NT was >3 at 4 hours post injection and increased continually in the whole observed period: T/NT ratio was >7 at 48 hours and >9 at 72 hours.

### 3.3. Biodistribution of ^**125**^I-anti-MIF McAb

As expected, the biodistribution of ^125^I-anti-MIF McAb showed the highest uptake and the lowest decrease in the inflammatory tissue. The activity in the blood was higher than the kidney, liver, spleen, heart, and lung. The change of activity in the heart and lung was the same as blood. Peak uptake in the kidney (0.2575±0.1640% ID/g, 0.2452±0.0612% ID/g, and 0.2909±0.0856% ID/g, respectively, in the *S. aureus*, *E. coli*, and turpentine oil group), liver (0.2271±0.1345% ID/g, 0.1682±0.0028% ID/g, and 0.1828±0.0955% ID/g, respectively in the *S. aureus*, *E. coli*, and turpentine oil group), spleen (0.1450±0.1621% ID/g, 0.0882±0.0799% ID/g, 0.1704±0.1351% ID/g, respectively in the *S. aureus*, *E. coli*, and turpentine oil group) occurred around 30 minutes, followed by gradual clearance over time. This indicated that the product of ^125^I-anti-MIF McAb was excreted from kidney or swallowed by reticuloendothelium of liver and spleen, resulting in deiodination.

### 3.4. Imaging of the inflammatory foci

**Figure 4:**
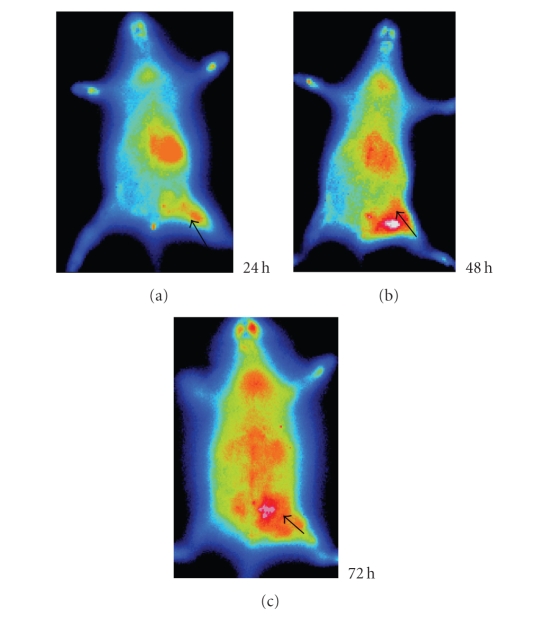
Serial images of *S. aureus* group at different times: the left thigh inflammation foci accumulated much more radioactivity of ^125^I-anti-MIF McAb; the highest was at 48 hours.

**Figure 5:**
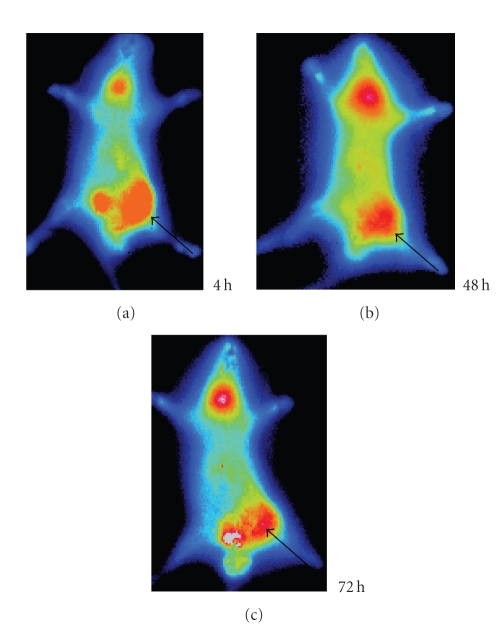
Serial images of turpentine group at different times: the left thigh inflammation lesion accumulated more radioactivity of ^125^I-anti-MIF McAb and was visualized clearly.

**Figure 6:**
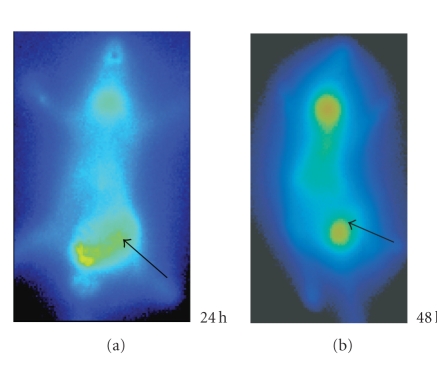
Serial images of *E. coli* group at different times: after 48 hours image of the left thigh inflammation foci was clear, but the amount of radioactivity was less than *S. aureus* or turpentine lesion.

Whole-body autoradiography showed that all inflammation foci could be visualized clearly from 24 hours after injection, but after 48 hours images were much clearer in accordance with the high T/NT ratio (Figures 4, 5, and 6). The radioactivity was the highest in *S. aureus* lesion, average in turpentine lesion, and the lowest in *E. coli* lesion.

## 4. DISCUSSION

The diagnosis of inflammatory processes is an important goal in medicine. In some cases the diagnosis is easy, based on the clinical history and the physical examination of patient. Other cases are more difficult to diagnose because they are asymptomatic or with nonspecific symptoms. Thus, nuclear medicine provides several techniques for in vivo detection of inflammatory processes. An expanding and even more interesting field of modern nuclear medicine is the development of radiolabelled receptor ligands, able to bind in vivo to specific receptors, allowing the noninvasive detection of specific cells and tissues.

Over the last decades, the imaging agents for inflammation have been developed rapidly: from nonbioactive chemicals to bioactive cell, and from macromolecule and antibody segments to peptides [16–20], and so on. Amongst the specific tracers, a new class of radiopharmaceuticals is represented by monoclonal antibodies (MoAb) [21–23]. The use of antibodies against surface granulocyte or lymphocyte antigens may improve the ability to detect inflammatory processes compared to the use of radiolabeled leukocytes.

The inflammatory response is rapid and includes secretion of cytokines and proinflammatory mediators that can induce an inflammatory response. MIF is believed to initiate inflammation by release of a number of proinflammatory cytokines including TNF-α, interleukin(IL)-1β, and IL-6, and to be implicated in the activation of T cells and macrophages. Recent reevaluation of MIF has suggested that MIF may be an important mediator of various inflammatory diseases, neutralization of MIF with either anti-MIF antibody or chemically derived inhibitors of MIF's enzymatic activity could be a valuable tool for treatment of various inflammatory disorders [24, 25].

In this study, we use the radioiodinated anti-MIF McAb to evaluate in vivo biology of MIF with the inflammation animal model in the BALB/c mice. Biodistribution studies of ^125^I-anti-MIF McAb in animals with *S. aureus*, *E. coli*, and turpentine oil inflammation indicated specific uptake in inflammatory tissues: T/NT was >3 at 4 hours post injection and increased continually up to >7 at 48 hours and >9 at 72 hours. High uptake in the kidney, liver, and spleen reflected that the product of ^125^I-anti-MIF McAb was excreted from kidney or swallowed by reticuloendothelium of liver and spleen, resulting in deiodination. Furthermore, serial images of whole-body autoradiography of ^125^I-anti-MIF McAb also demonstrated clear delineation of the inflammatory foci. All foci of the inflammation could be visualized clearly from 24 hours post injection.

This study demonstrates the ability of radioiodinated anti-MIF McAb to measure in vivo inflammatory events represented by high expression of MIF and suggests that radiolabeled anti-MIF McAb warrants further investigation as a potential inflammation-seeking agent for imaging to detect inflammatory disorders.
